# Automatic detection of intracranial aneurysms in 3D-DSA based on a Bayesian optimized filter

**DOI:** 10.1186/s12938-020-00817-9

**Published:** 2020-09-15

**Authors:** Tao Hu, Heng Yang, Wei Ni, Yu Lei, Zhuoyun Jiang, Keke Shi, Jinhua Yu, Yuxiang Gu, Yuanyuan Wang

**Affiliations:** 1 Department of Electronic Engineering, Fudan University, Shanghai, 200433 China; 2 Department of Neurosurgery, Huashan Hospital, Fudan University, Shanghai, 200040 China

**Keywords:** Aneurysm detection, Multiscale filter, Bayesian optimization, Adaptive thresholding

## Abstract

**Background:**

Intracranial aneurysm is a common type of cerebrovascular disease with a risk of devastating subarachnoid hemorrhage if it is ruptured. Accurate computer-aided detection of aneurysms can help doctors improve the diagnostic accuracy, and it is very helpful in reducing the risk of subarachnoid hemorrhage. Aneurysms are detected in 2D or 3D images from different modalities. 3D images can provide more vascular information than 2D images, and it is more difficult to detect. The detection performance of 2D images is related to the angle of view; it may take several angles to determine the aneurysm. As the gold standard for the diagnosis of vascular diseases, the detection on digital subtraction angiography (DSA) has more clinical value than other modalities. In this study, we proposed an adaptive multiscale filter to detect intracranial aneurysms on 3D-DSA.

**Methods:**

Adaptive aneurysm detection consists of three parts. The first part is a filter based on Hessian matrix eigenvalues, whose parameters are automatically obtained by Bayesian optimization. The second part is aneurysm extraction based on region growth and adaptive thresholding. The third part is the iterative detection strategy for multiple aneurysms.

**Results:**

The proposed method was quantitatively evaluated on data sets of 145 patients. The results showed a detection precision of 94.6%, and a sensitivity of 96.4% with a false-positive rate of 6.2%. Among aneurysms smaller than 5 mm, 93.9% were found. Compared with aneurysm detection on 2D-DSA, automatic detection on 3D-DSA can effectively reduce the misdiagnosis rate and obtain more accurate detection results. Compared with other modalities detection, we also get similar or better detection performance.

**Conclusions:**

The experimental results show that the proposed method is stable and reliable for aneurysm detection, which provides an option for doctors to accurately diagnose aneurysms.

## Background

Intracranial aneurysm is a serious life-threatening cerebrovascular disease and usually occurs around the arteries at the base of the brain called the Circle of Willis. The worldwide incidence of aneurysms is approximately 3% [1]. In a cross-sectional study in China, 7% of adults between the ages of 35 and 75 years had an aneurysm detected on widespread screening with brain magnetic resonance angiography (MRA) [2]. Subarachnoid hemorrhage (SAH), which results from the rupture of an intracranial aneurysm, is a devastating event associated with high rates of mortality (40–50%) and morbidity, while only 40% of SAH patients recover to reach independent status [3, 4]. Aneurysm rupture is a rare event; nevertheless, early detection is essential for its prevention. With early detection, the growth of aneurysms can be halted by interventional or surgical treatment [5]. Intracranial aneurysms are usually asymptomatic before rupture and are often found incidentally [6]. The increasing use of medical imaging devices has led to an increased diagnosis rate of unruptured intracranial aneurysms. Image modalities that are used in aneurysm diagnosis usually include computed tomography angiography (CTA), magnetic resonance angiography (MRA), and digital subtraction angiography (DSA). These imaging techniques can adequately show the location, size and shape of aneurysms and help doctors make reasonable treatment plans [7]. For some small intracranial aneurysms, CTA and MRA diagnosis performance is not as good as DSA, which has been used as the ground truth for aneurysm diagnosis [8, 9].

Detection of aneurysms can help doctors to improve the accuracy of aneurysm diagnosis, allowing them to take effective measures for developing a corresponding treatment to reduce the risk of arachnoid hemorrhage. Generally, aneurysms are detected from 2D or 3D images. Different detection systems have been tested with different angiographic modalities [10]. Many studies have shown some progress in the detection of aneurysms on 2D-DSA. Sulayman et al. [11] proposed a semiautomatic detection algorithm that combined image processing and machine learning and achieved 89.5% sensitivity and 81% positive predictive value on 2D-DSA. Jin et al. [12] proposed a novel, fully automated detection and segmentation on 2D-DSA time series images. They combined a deep learning model (U-net) with long short-term memory (LSTM) and obtained an aneurysm detection sensitivity of 89.3%. Rahmany et al. [13] separated blood vessels from the background, and further processed the vessel regions to detect aneurysms by integrating MSER, SURF and SIFT feature descriptors. The sensitivity of their method was 100%, but only the data from three patients were analyzed. Other detection methods have only been evaluated for a single patient; these methods are generally based on traditional image processing [14, 15].

In addition to 2D-DSA, aneurysm detection systems have also been developed for using on 3D-MRA and 3D-CTA. Hanaoka et al. [16] extracted a novel feature named HoTPiG (Histogram of Triangular Paths in Graph) from 3D-MRA. Then, they used a traditional machine learning method to detect aneurysms, and the results showed a sensitivity of 89.2%. Sichterman et al. [17] first preprocessed 3D-MRA with different methods and then used a neural network that consisted of two pathways with 11 layers to detect intracranial aneurysms; the sensitivity reached 90%. Hentschke et al. [18] combined both low-level and high-level features, which were used to classify and detect aneurysms on MRA and CTA. They achieved a sensitivity higher than 93% for three modalities. After that, Hentschke et al. [19] detected aneurysms with three angiographic modalities (CE-MRA, TOF-MRA, CTA) by combining the features of shape information, spatial information and probability information. The true positives could be distinguished with a linear discriminant function (LDF), reaching a sensitivity of 95% for the three modalities, and 93% of aneurysms that were smaller than 5 mm were found. According to the local and global geometric characteristics of the aneurysm, Zhou et al. [20] used a pretrained deep learning model to detect aneurysms on different 3D modalities and obtained 94.7% accuracy and 94.8% sensitivity. Yang et al. [21] proposed a fully automated computer-aided detection (CAD) scheme for detecting aneurysms on 3D MRA. First, they used two methods to extract points of interest (POI) and then reduced false positives according to feature analysis, obtaining a sensitivity of 96%, but only 91% of aneurysms that were smaller than 5 mm were found.

In the clinical diagnosis of aneurysms, the detection performance for 2D images depends on the selected viewing angle. However, multiple aneurysms may not be all observed at one angle. 3D images can provide more information than 2D images, and its detection is more difficult. As the gold standard for the diagnosis of intracranial aneurysms, DSA is an optimal choice for aneurysm detection. Detection on 3D-DSA has more clinical value than on other 3D modalities.

The enhanced filter based on Hessian matrix has great potential in computer-aided diagnosis. Some multiscale enhancement filters were proposed to enhance specific shape structures [22–25]. This idea can also be used in aneurysm detection, but one of the difficulties was in setting the parameters for the multiscale filters. In previous studies, the parameters for multiscale filters were set manually. Therefore, it will be much convenient if these parameters could be searched automatically. For the search of hyperparameters, the common search methods include Grid search, Random search, Genetic algorithm and Bayesian optimization [26]. In fact, grid search and random search are very common and general methods; their search ability is not good as genetic algorithm. However, genetic algorithm also has some defects, such as poor local search ability, long time consuming and slow searching speed [27]. The advantage of Bayesian optimization is to use Gaussian process to adjust the parameters, which will consider the previous search information and constantly update the prior knowledge [28]. Moreover, Bayesian optimization has fewer iterations and faster speed, and it is still stable for non-convex problems [29]. In this study, we proposed an automatic detection of intracranial aneurysms on 3D-DSA based on a Bayesian optimized filter.

## Results

In the evaluated data, there were 165 aneurysms in 145 patients, among whom 127 patients had one aneurysm, 16 patients had two aneurysms, and 2 patients had three aneurysms. The response of the enhancement filter was visualized by rendering technology. After the optimal scale parameters were obtained by Bayesian optimization, the proposed filter was used to detect the aneurysms.

**Fig. 1:**
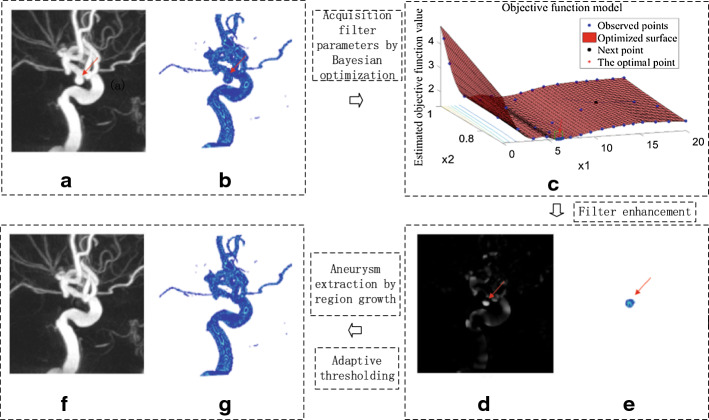
The first aneurysm detection process. **a** Display the original image with MIP. **b** Display the original image with 3D rendering. **c** The process of searching parameters by Bayesian optimization for the first aneurysm detection. **d** The first aneurysm detected is shown by MIP. **e** The first aneurysm detected is shown by 3D rendering. **f** Image after removing the first aneurysm is shown by MIP. **g** Image after removing the first aneurysm is shown by 3D rendering

**Fig. 2:**
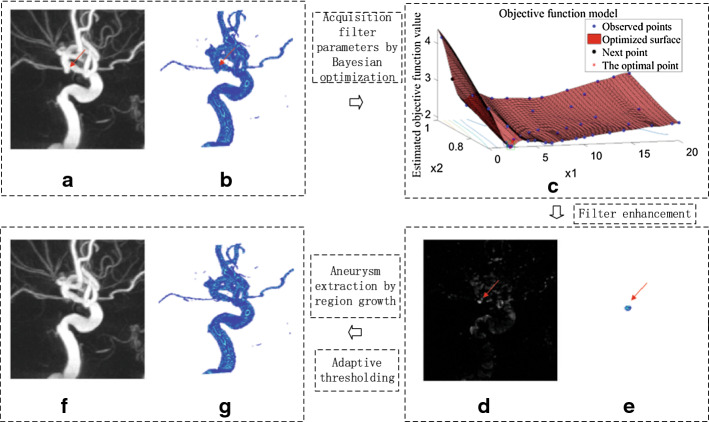
The second aneurysm detection process. **a** Image after removing the first aneurysm is shown by MIP. **b** Image after removing the first aneurysm is shown by 3D rendering. **c** The process of searching parameters by Bayesian optimization for the second aneurysm detection. **d** The second aneurysm detected is shown by MIP. **e** The second aneurysm detected is shown by 3D rendering. **f** Image after removing the second aneurysm is shown by MIP. **g** Image after removing the second aneurysm is shown by 3D rendering

All 3D-DSA data were analyzed with the same method. These data may or may not contain aneurysms, and there may be one or multiple aneurysms in the data. In clinical diagnosis, although most patients have only one aneurysm, multiple aneurysms also occur occasionally. In our method, when the detected target was an aneurysm, it was removed, and then the steps were repeated in the remaining image to continue detection. In the second round of detection, another set of optimal parameters was obtained. These parameters were substituted into the filter and used to continue to detect aneurysms in the remaining image. In this way, two aneurysms in a patient could be detected. When there were more than two aneurysms in a patient, the detection method was repeated again. Figures 1 and 2 illustrate the detection results of two aneurysms in a single patient.

Figure 1 shows the first aneurysm detection process. The maximum intensity projection (MIP) and 3D visual rendering were used to display the blood vessels and aneurysms, respectively. In Fig. 1, the red arrow indicates the first aneurysm. Figure 1a, b shows the image to be detected.

Figure 1c shows the Bayesian optimization process for finding the parameters of the aneurysm filter, where ×1 and ×2 represent the two parameters $\left( {{\text{s}},\tau } \right)$, respectively, of the filter. The ordinate represents the target function value corresponding to each group of parameters, the blue point represents the point that has been found in each search process, and the black point is the next group of parameter points found by model calculation in the Bayesian optimization process. Since the number of calculations was set to 50 in advance, there are 50 points in the graph. In the final result, the point with the lowest objective function value is the optimization point, which is represented by a red asterisk.

When the optimal parameters were obtained by Bayesian optimization, these parameters were substituted into the filter for aneurysm detection. Figure 1d, e shows the filtered image. As seen from the figure, the aneurysm was obviously enhanced, and other areas, including blood vessels, were suppressed to dark pixel values. In Fig. 1f, g, the detected aneurysm was removed by region growth. According to the detection rules, if the first target was detected as an aneurysm, it was removed, and then the detection steps were repeated until the target was not an aneurysm.

Figure 2 shows the Bayesian optimization process and detection results for the second aneurysm. The detection process and image display were the same as the first aneurysm. Also, Fig. 2a, b shows the image to be detected. Figure 2c shows the Bayesian optimization procedure for detection of the second aneurysm. Figure 2d, e shows the filtered image, in which the red arrow indicates the detected aneurysm. According to the same rule used for the first aneurysm, the second aneurysm was also extracted by region growth. After the first and second aneurysms were detected, the detection process was continued, as shown in Fig. 2f, g. When no more aneurysms were detected, the detection process was stopped.

The filter can enhance not only spherical aneurysms but also aneurysms that slightly deviate from a spherical structure. In our method, mean value of the filtering response of the detected target ($V_{\text{mean}}$) was used to detect the aneurysm. As the threshold was gradually changed, we could determine whether the detected target was an aneurysm by comparing the value of $V_{\text{mean}}$ with the threshold. Maximum value of the filtering response of the detected target ($V_{ \hbox{max} }$) was also used for comparison, and the detection method was the same as that of $V_{\text{mean}}$. During filter processing, since the response of the aneurysm region is uniform, it is theoretically better to use the mean value. The diagnosis of two neuroradiologists served as a reference standard. The performance of the proposed method was objectively evaluated by precision, recall, and *F*1-score, which were defined as:

1 $$\begin{aligned} {\text{Precision}} & = \frac{\text{TP}}{{{\text{FP}} + {\text{TP}}}}, \\ {\text{Recall}} & = \frac{\text{TP}}{{{\text{TP}} + {\text{FN}}}}, \\ F1{\text{ - score}} & = \frac{{2*{\text{Precision*Recall}}}}{{{\text{Precision}} + {\text{Recall}}}}, \\ \end{aligned}$$

where $${\text{TP}}$$, $${\text{TN}}$$, $${\text{FP}}$$, $${\text{FN}}$$ are the number of true-positive aneurysms, number of true-negative aneurysms, number of false-positive aneurysms, and number of false-negative aneurysms, respectively. Recall can also be called sensitivity, which is a measure of coverage, and is the proportion true positives among all actual aneurysms. The precision–recall (PR) curve can be obtained by plotting recall on the horizontal axis and precision on the vertical axis. According to the values of precision and recall, the *F*1-score can be calculated; when the value of *F*1-score is maximized, the detection performance is regarded as the best. It is also possible to calculate the false positive rate, which is defined as $${\text{FPR}} = {\raise0.7ex\hbox{${\text{FP}}$} \!\mathord{\left/ {\vphantom {{\text{FP}} {\left( {{\text{FP}} + {\text{TN}}} \right)}}}\right.\kern-0pt} \!\lower0.7ex\hbox{${\left( {{\text{FP}} + {\text{TN}}} \right)}$}}$$. The receiver operator characteristic (ROC) curve can be drawn from the sensitivity and false positive rate. This curve can also reflect the performance of detection, more specifically by calculating the area under the curve (AUC).

**Fig. 3:**
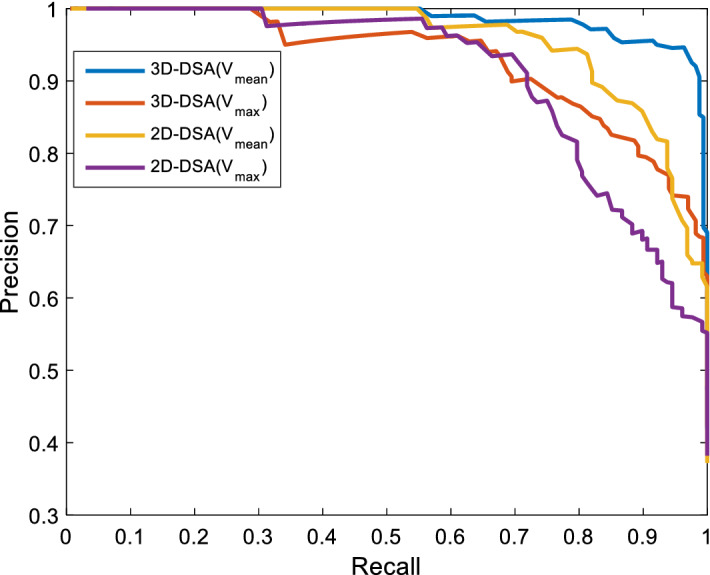
The PR curve of the detection results

**Fig. 4:**
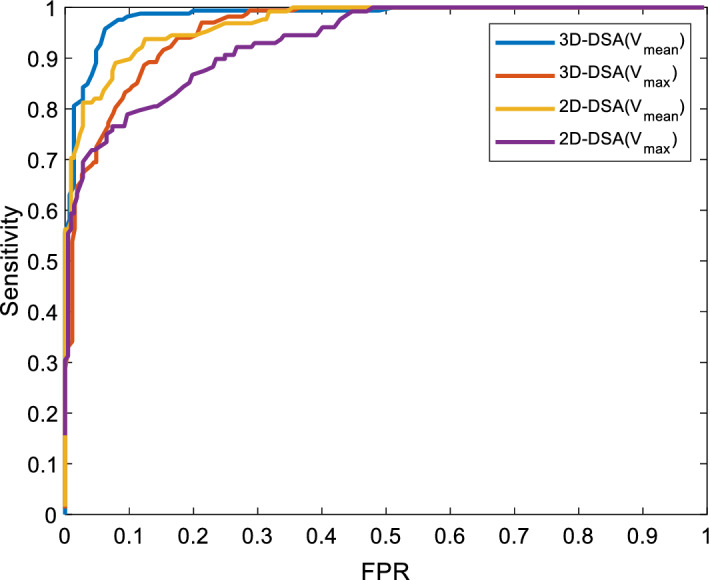
The ROC curve of the detection results

We also detected aneurysms on 2D-DSA for comparison. First, we selected a view angle that clearly showed an aneurysm on 2D-DSA for each patient and then detected the aneurysm with the method described in this paper, only changing the dimension from 3D to 2D. When the target was extracted, the maximum or mean value of the response of the target was compared with the threshold. When the threshold was gradually changed, different values of precision and recall could be obtained. In this way, a series of different detected results were obtained. The detection process can be drawn as a PR curve and as a ROC curve, which are shown in Figs. 3 and 4, respectively. To increase the experimental contrast, each patient was also assessed by a human. The best detection results are listed in Table 1.

**Table 1:** Detection results of 3D-DSA and 2D-DSA

	Precision (%)	Recall (%)	*F*1-score (%)	AUC
3D-DSA $(V_{\text{mean}} )$	94.6	96.4	95.5	0.98
3D-DSA $(V_{\hbox{max} } )$	82.1	88.7	85.2	0.94
2D-DSA $(V_{\text{mean}} )$	85.8	89.8	87.8	0.95
2D-DSA $(V_{\hbox{max} } )$	87.2	75.0	80.6	0.91
2D-observed	94.5	93.3	93.9	–

**Fig. 5:**
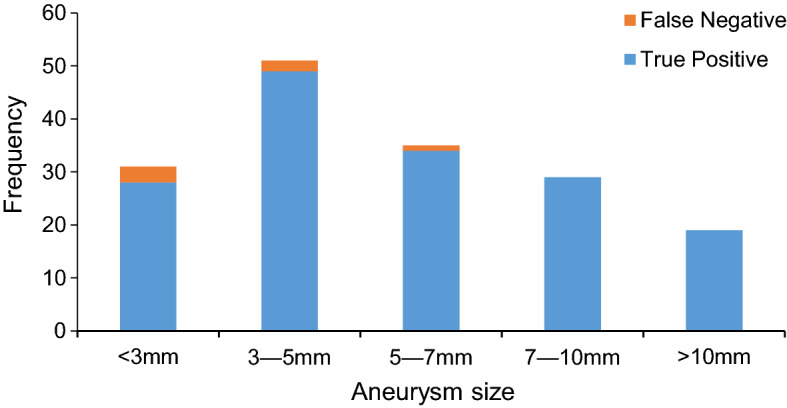
Histogram of aneurysm size and detection results

As shown in the PRC and ROC curves, 3D-DSA $(V_{\text{mean}} )$ had the best performance and conformed to the theoretical analysis. From the experimental results, we obtained a precision of 94.6% and a recall of 96.4%. When the sensitivity was 96.4%, the false-positive rate was 6.2%. In our experiment, 6 of 165 aneurysms remained undetected by the proposed method. A histogram of aneurysm size and detection frequency on 3D-DSA is shown in Fig. 5. From the detection results, 5 of the false-negative aneurysms were under 5 mm, and the detection rate for small aneurysms (< 5 mm) was thus (82 − 5)/82 ≈ 93.9%. The mean size of the 6 undetected aneurysms was 3.63 mm, and four of them were not detected by humans on 2D-DSA. Because of the nonuniformity of the filter response, the position of some vascular protrusions could also be enhanced very well; so, the performance using the maximum response value as the threshold value was not as good as that of the mean response value. On 2D-DSA, due to the limitation of the 2D image perspective, a total of 163 observed targets were regarded as aneurysms, of which 154 were true positive, resulting in a recall of 93.3%. In the automatic detection of aneurysms, overlapping curved vessels and aneurysmal areas are easily confused in 2D-DSA; therefore, more false positives are obtained. The experiment shows that the performance of aneurysm detection in 3D-DSA is better than that in 2D-DSA.

## Discussion

In this section, we also compared with other aneurysm detection algorithms. According to the known literature, many aneurysm detection systems were carried on the modalities of 2D-DSA, 3D-MRA/MRI and 3D-CTA. Since the evaluation criteria of each algorithm were different, we selected some criteria for comparison, and the results are shown in Table 2.

**Table 2:** Performance comparison with other aneurysm detection methods in different data sets

Algorithms	Modality	*N*	*F*1-score (%)	SE (%)
Sichterman et al. [17]	3D-MRA	85	–	87.0
Sulaymana et al. [11]	2D-DSA	19	–	89.5
Jin et al. [12]	2D-DSA	493	–	89.3
Hanaoka et al. [16]	3D-MRA	300	–	89.2
Zhou et al. [20]	3D-RA + 3D-MRI	121	94.7	94.8
Hentschke et al. [19]	3D-MRA + 3D-CTA	66	–	95.0
Proposed	3D-DSA	145	95.5	96.4

*N*: number of cases; SE: sensitivity

When comparing the performance of several other algorithms, the results of our method are superior in some indicators due to differences in data sets and evaluation criteria. As can be seen from Table 2, our method achieved the highest sensitivity. Sulayman et al. [11] combined the traditional feature extraction and machine learning method, 89% detection sensitivity was obtained in 19 cases. Jin et al. [12] used deep learning to detect aneurysms in more 2D-DSA cases; the detection sensitivity was the same as Sulayman’s method. They also combined with image segmentation and got a lower dice coefficient. Clemens et al. [19] obtained 95% sensitivity by traditional machine learning, and the sensitivity of small aneurysms detection was 93%, while our result was 93.9%. The *F*1-score obtained by Zhou et al. [20] through deep learning was similar to our proposed method, but we had higher sensitivity. These aneurysm detection systems usually use traditional hand-crafted feature machine learning methods or other deep learning methods, and they were detected on the same dimension of image modality. In this paper, we used Bayesian optimization to automatically find filter parameters, and detected aneurysms on both 2D-DSA and 3D-DSA. As far as we know, it is the first time that Bayesian optimization is used in the automatic search for filter parameters, and based on the theory of Hessian matrix and traditional image processing, we get good detection performance.

We chose DSA instead of other neuroimaging modalities, such as MRA or CTA, for aneurysm intracranial detection because DSA provides maximum contrast between cerebrovascular and surrounding tissues and DSA remains the most effective modality in the diagnosis of cerebrovascular diseases. Compared to 3D images, 2D images are limited by different, constrained view angles, the inability to see some of the aneurysms in some sections, and the interference from overlapping parts of the bending of blood vessels. However, these problems do not affect 3D images, which can provide more information for detection.

This paper presented a novel framework that performed structure enhancement and Bayesian optimization of brain DSA to accurately identify intracranial aneurysms. The methodology presented in the preceding sections efficiently solved the most significant research questions through comprehensive investigation of filter processing and automatic filter parameter adjustment. This method was based on the principle that eigenvalues of the Hessian matrix can enhance objects of different shapes. A filter based on the eigenvalues of the Hessian matrix was constructed, and the optimal parameters were found by Bayesian optimization. By enhancing spherical structures and suppressing other structures, aneurysms could be detected. The proposed method is reliable for the detection of intracranial aneurysms in a patient one at a time.

However, our system also has limitations. The detection of small aneurysms is still very difficult because of the overlap of vascular tissue. The system also generated some false positives, although they could be easily distinguished by humans. Computer-aided diagnosis is helpful for doctors to diagnose aneurysms accurately and analyze some of their properties, such as aneurysm rupture. In the future research, we can improve the detection filter and aneurysm extraction rules to increase the detection sensitivity of small aneurysms. We can also combine the three-dimensional deep learning to obtain better detection performance.

## Conclusions

In this paper, we proposed an automatic aneurysm detection method based on Bayesian optimization filter in 3D-DSA. First, we constructed a multiscale enhancement filter based on the attribute of Hessian matrix, and then used Bayesian optimization to automatically search for the optimal detection parameters of the aneurysm filter. We found that the mean response of the filter was a good discriminating parameter, and the adaptive threshold method can be used to determine whether the enhanced target was an aneurysm. When the aneurysm was detected, region growth method was used to remove the aneurysm. The detection was continued in the remaining images, and each aneurysm corresponds to a set of optimal detection parameters. The experimental results show that our method was superior to other methods.

This method was evaluated on 145 patients with 165 intracranial aneurysms, and the quantitative assessment showed good performance of aneurysm detection. We obtained a detection precision of 94.6%, and a sensitivity of 96.4% with a false-positive rate of 6.2%. The *F*1-score and AUC can reach 95.5% and 0.98, respectively; only 6 aneurysms remained undetected. The analysis was carried out in MATLAB (R2018b).

The proposed method can be used to assist doctors in the diagnosis of aneurysms, which is a stable and reliable detection method. Therefore, we hope that it can give a technical option to improve the accuracy of intracranial aneurysms detection. The major contributions can be summarized as follows:

1. We carried out aneurysm detection on 3D-DSA; compared with other 3D modalities, this modality can provide more complete aneurysm information and reduce the rate of missing aneurysm detection.
2. We proposed a multiscale aneurysm enhancement filter and established the relationship between aneurysm detection and Bayesian optimization.
3. We used Bayesian optimization to automatically search for the detection parameters of the aneurysm filter, where each aneurysm corresponded to a set of optimal filter parameters.
4. For multiple aneurysms, we used an iterative detection strategy and an adaptive threshold based on the region growth method to extract the aneurysms.

## Materials and methods

### Materials

The original angiography data were obtained from the Department of Neurosurgery, Huashan Hospital, Fudan University. This dataset consisted of information from 145 patients who had data from both 3D-DSA and corresponding 2D-DSA. The image pixel spacing of DSA was 0.4–0.6 mm. Aneurysms from each patient were studied in this paper. The baseline characteristics of the patients are shown in Table 3. “Single” denotes patients with only one aneurysm, and “Multiple” denotes patients with two or more aneurysms; among the “Multiple” patients, 18 had a total of 38 aneurysms. In the following subsection, the proposed method will be described in detail.

**Table 3:** Baseline characters of patients in this study

Parameters	Single (*n* = 127)	Multiple (*n* = 18)
Sex (number)		
Male	43	7
Female	84	11
Age (mean ± standard deviation)		
Male	57.8 ± 11.6	52.6 ± 11.2
Female	56.9 ± 10.7	57.6 ± 11.3
Size (mm)		
< 3	20	16
3–6	61	15
6–10	29	4
> 10	17	3
Location		
ACoA	7	4
MCA	22	6
PCoA	8	5
ICA	84	20
ACA	6	3

*ACoA* anterior communicating artery, *ACA* anterior cerebral artery, *ICA* internal carotid artery, *MCA* middle cerebral artery, *PCoA* posterior communicating artery

### Methods

**Fig. 6:**
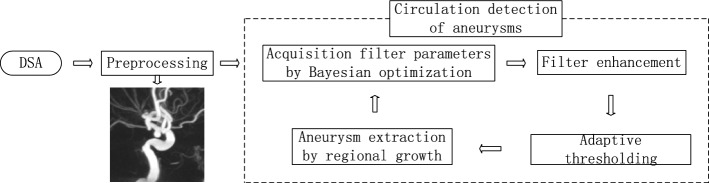
The scheme of the proposed algorithm

Figure 6 shows a flowchart of the proposed method. First, the original images were preprocessed. Then, we constructed a filter based on the eigenvalues of the image voxel Hessian matrix and automatically determined the filter parameters through Bayesian optimization. The filter is capable of enhancing the aneurysm area and inhibiting the other tissues of the blood vessels. In the experiment, we found that the mean value of the filter response of the detected target can be used to screen for aneurysms. When an aneurysm is detected, it can be removed by region growth. We can repeat the previous steps to detect any aneurysms in the remaining image. Generally, the detailed process of the proposed computer-aided aneurysm detection system includes five steps: (1) preprocessing; (2) optimal filter parameter determination by Bayesian optimization; (3) filter enhancement; (4) adaptive thresholding; (5) aneurysm extraction.

### Preprocessing

**Fig. 7:**
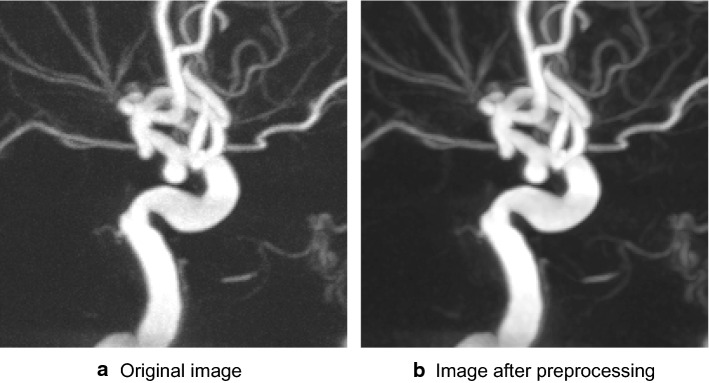
Image preprocessing process

Given the existence of some noise in the image, a $3 \times 3 \times 3$ median filter was used to remove the noise. Then, the image pixel values were normalized to the range 0–1. The image preprocessing process is shown in Fig. 7.

### Optimal filter parameter determination by Bayesian optimization

Enhancement filters can enhance certain structures in an image. Previous studies [23–25] have proposed various multiscale filters based on the second derivative of image intensity. Given that the shape of the aneurysm can be approximated as a sphere, we can regard the detection of the aneurysm as a filtering process using a spherical structure filter. Since the size of aneurysms is variable, it is important to introduce a scale parameter that varies adaptively. Let the intensity of the coordinate point $x = \left[ {x_{1} ,x_{2} ,x_{3} } \right]^{\text{T}}$ in a 3D image be $I\left( x \right)$. To analyze the local behavior of an image $I\left( x \right)$, Taylor expansion was carried out as follows:

2 $$I\left( {x_{0} + \varepsilon x_{0} ,s} \right) \approx I\left( {x_{0} ,s} \right) + \varepsilon x_{0}^{\text{T}} \nabla_{0,s} + \varepsilon x_{0}^{\text{T}} H_{0,s} \varepsilon x_{0}^{\text{T}} ,$$

where $\nabla_{0,s}$ and $H_{0,s}$ are gradient vectors and the Hessian matrix of images at point $x_{0}$, respectively, and *s* denotes the scale parameter. A scale space is introduced, and the second-order derivative of $I\left( x \right)$ is a $3 \times 3$ Hessian matrix, which can be defined as a convolution with derivatives of Gaussians:

3 $$H = s^{2} I\left( x \right)*\left[ {\begin{array}{*{20}c} {\frac{{\partial^{2} G\left( {x,s} \right)}}{{\partial x_{1}^{2} }}} & {\frac{{\partial^{2} G\left( {x,s} \right)}}{{\partial x_{1} \partial x_{2} }}} & {\frac{{\partial^{2} G\left( {x,s} \right)}}{{\partial x_{1} \partial x_{3} }}} \\ {\frac{{\partial^{2} G\left( {x,s} \right)}}{{\partial x_{2} \partial x_{1} }}} & {\frac{{\partial^{2} G\left( {x,s} \right)}}{{\partial x_{2}^{2} }}} & {\frac{{\partial^{2} G\left( {x,s} \right)}}{{\partial x_{2} \partial x_{3} }}} \\ {\frac{{\partial^{2} G\left( {x,s} \right)}}{{\partial x_{3} \partial x_{1} }}} & {\frac{{\partial^{2} G\left( {x,s} \right)}}{{\partial x_{3} \partial x_{2} }}} & {\frac{{\partial^{2} G\left( {x,s} \right)}}{{\partial x_{3}^{2} }}} \\ \end{array} } \right],$$

where $$G\left( {x,s} \right) = \left( {2\pi s^{2} } \right)^{{{\raise0.7ex\hbox{${ - 3}$} \!\mathord{\left/ {\vphantom {{ - 3} 2}}\right.\kern-0pt} \!\lower0.7ex\hbox{$2$}}}} \exp \left( { - \frac{{x^{\text{T}} x}}{{2s^{2} }}} \right)$$ is a 3D Gaussian function, and $$*$$ denotes convolution. Assume the three eigenvalues of the Hessian matrix in 3D-DSA are $$\lambda_{1} ,\lambda_{2} ,\lambda_{3}$$, and they are sorted according to their magnitude $$\left| {\lambda_{1} } \right| \le \left| {\lambda_{2} } \right| \le \left| {\lambda_{3} } \right|$$. The eigenvalues of the Hessian matrix can enhance the structure of different shapes. For spherical objects, the relationship between the magnitude and signs of the eigenvalues with the desired enhancement pattern is shown in Table 4 [30].

**Table 4:** Enhancement pattern in 2D and 3D (H = high, L = low, ± indicates the sign of eigenvalue, $\left| {\lambda_{1} } \right| \le \left| {\lambda_{2} } \right| \le \left| {\lambda_{3} } \right|$)

2D		3D			Shape (enhancement)
$\lambda_{1}$	$\lambda_{2}$	$\lambda_{1}$	$\lambda_{2}$	$\lambda_{3}$	
H−	H−	H−	H−	H−	Spherical (bright)
H+	H+	H+	H+	H+	Spherical (dark)

The magnitude and signs of the eigenvalues of the Hessian matrix reflect the specific shape of the structure, and a filter that is composed of these eigenvalues can enhance objects of different shapes. Bright spherical structures on a dark or bright background can be represented by negative and positive eigenvalues, respectively. In addition to the properties shown in Table 2, when $\left( {\lambda_{1} \approx \lambda_{2} \approx \lambda_{3}^{{\left| {\lambda_{1,2,3} } \right|}} \le 0} \right)$, the spherical structure will also be enhanced. Based on the Hessian matrix eigenvalue attributes of 3D images, different filters can be constructed to enhance a specific shape structure. In this paper, inspired by the method proposed by Jerman [31], we used an enhancement filter that combines these eigenvalues to detect intracranial aneurysms as follows:

4 $$B_{\text{p}} = \left( {\exp^{{B_{1} }} - 1} \right)/\left( {\exp - 1} \right),$$

$$B_{1} = \frac{{2\left( {\lambda_{1}^{2} \lambda_{\rho } \left( {\frac{3}{{2\lambda_{1} + \lambda_{\rho } }}} \right)^{3} + \frac{{\lambda_{1}^{2} }}{{\left| {\lambda_{\rho } } \right|}} + \sqrt {\lambda_{1} *\lambda_{\rho } } } \right)}}{3},$$

5 $$\lambda_{\rho } = \left\{ {\begin{array}{*{20}c} {\lambda_{3} \quad {\text{if}}\quad \lambda_{3} < \tau *\hbox{min} \left( {\lambda_{3} \left( {x,s} \right)} \right)} \\ {\tau *\hbox{min} (\lambda_{3} \left( {x,s} \right)) \quad {\text{otherwise}}} \\ \end{array} } \right.,$$

where $\lambda_{3} \left( {x,s} \right)$ denotes $\lambda_{3} (x)$ at scale *s*; the minimum of all $\lambda_{3} \left( {x,s} \right)$ is computed to find the eigenvalue with the highest magnitude. The value of $\tau$ determines the response intensity of the filter. To obtain the maximum filter response value, we need to compare the eigenvalues for each point $x$ at scale *s* on the image. The value of *s* depends on the size of the aneurysm. The values of $s$ and $\tau$ vary within certain ranges; $s$ varies from 0 to 20, and $\tau$ varies from 0.7 to 1. For aneurysm detection, the goal is to find a set of parameters $\left( {s,\tau } \right)$ that maximize the value of function $B_{\text{p}}$. The exponential function in $B_{\text{p}}$ is used to increase the contrast between light and dark. In our experiment, we can find the maximum value of function $B_{\text{p}}$ quickly with Bayesian optimization, which automatically finds the parameters. The second eigenvalue $\lambda_{2}$ does not provide information for distinguishing spherical shapes from other shapes; so, this value is not used to construct the filter.

To obtain the maximum value of the filter, the parameters need to be adjusted repeatedly, and each set of parameters should be calculated for comparison because different scale parameters may enhance different shape and size structures. Bayesian optimization can automatically and quickly find the optimal set of parameters without artificially selecting or setting any of them [32]. Assume a set of hyperparameters $$Z = \left\{ {z_{1} ,z_{2} , \ldots z_{n} } \right\}$$, where each $$z_{i} = \left( {s_{i} ,\tau_{i} } \right), i = 1,2,3 \ldots n,$$ denotes a set of filter parameters in our experiment, and a corresponding relation between the set of hyperparameters and the final loss function $$f\left( Z \right)$$. If there is a function $$f:Z \to R$$, using the reciprocal of the proposed filter $$\left( {{\raise0.7ex\hbox{$1$} \!\mathord{\left/ {\vphantom {1 {B_{\text{p}} }}}\right.\kern-0pt} \!\lower0.7ex\hbox{${B_{\text{p}} }$}}} \right)$$ as the loss function in our method, the goal is to find $$Z \in R$$ that makes:

6 $$z^{*} = \underbrace {\arg \hbox{min} f\left( z \right)}_{z \in Z}.$$

The flow chart of the Bayesian algorithm is as follows:
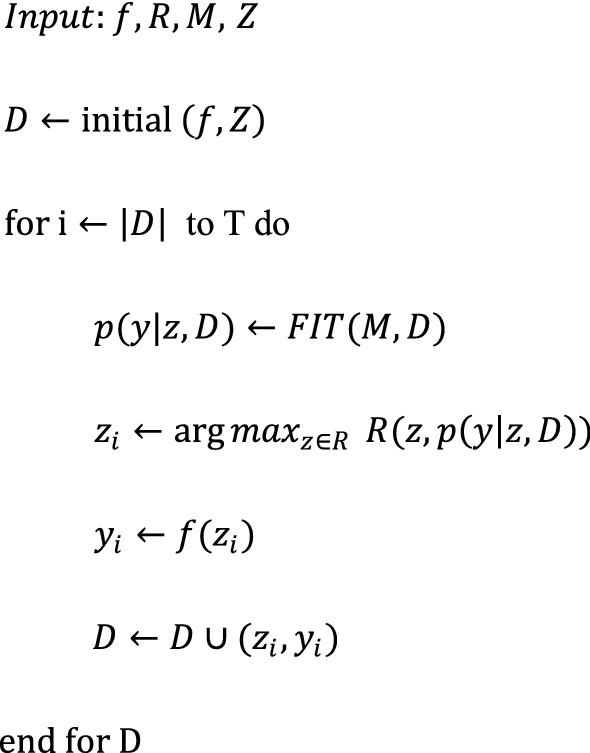


where $f$ is the form of the loss function; $Z$ are the filter parameters; $R$ is the acquisition function; $M$ is the hypothesis module of the input data; *y* is the value of the loss function; and *T* is the number of iterations. Since each calculation requires many computing resources, the number of iterations was set to 50 in this paper. The specific process of Bayesian optimization calculation is as follows:

Step 1: Obtain the initialized dataset $D = \left( {z_{1} ,y_{1} } \right), \ldots ,\left( {z_{n} ,y_{n} } \right)$;
Step 2: Calculate the concrete form of the model *M* function from the assumed model $M$ and initial dataset $D$;
Step 3: Acquire the next data point through the acquisition function $R$;
Step 4: Calculate the new value $f\left( {z_{i} } \right)$ and update the dataset

The input data model is assumed to follow a Gaussian distribution; then, $f\sim {\text{GP}}\left( {u,K} \right)$ (GP: Gaussian process, $u$: mean, $K$: covariance kernel), so the new prediction data also obey a normal distribution:

$$p\left( {y |z,D} \right) = N(y|\hat{u},\hat{\sigma }^{2} )$$

To obtain the concrete expression of function $p\left( {y |z,D} \right)$, $y,\hat{u},\hat{\sigma }^{2}$ should be computed first; they are defined as

7 $$y = (y_{1} \ldots y_{i} )^{\text{T}} ,$$

8 $$\hat{u} = k\left( z \right)^{\text{T}} (K + \sigma_{n}^{2} |)^{ - 1} y,$$

9 $$\hat{\sigma }^{2} = k\left( {z,z} \right) - k\left( z \right)^{\text{T}} (K + \sigma_{n}^{2} |)^{ - 1} k\left( z \right).$$

To avoid falling into a local minimum, the acquisition function $R$ is defined as:

10 $$\begin{aligned} a_{\text{EI}} \left( z \right) & = \int\limits_{ - \infty }^{{f^{\prime}}} {\left( {f^{\prime} - f} \right)N\left( {f;u\left( z \right),k\left( {z,z} \right)} \right){\text{d}}f} \\ & = \left( {f^{\prime} - u\left( z \right)} \right)\varPhi \left( {f^{\prime};u\left( z \right),k\left( {z,z} \right)} \right) + k\left( {z,z} \right)N\left( {f^{\prime};u\left( z \right),k\left( {z,z} \right)} \right), \\ \end{aligned}$$

where $f^{\prime} = \hbox{min} f\left( z \right)$ and $u\left( z \right) = \hbox{max} \left( {0,f^{\prime} - f\left( z \right)} \right)$. Finding the maximum absolute value of the difference between $f^{\prime}$ and $f\left( z \right)$ as a reward, $u\left( {\text{z}} \right)$ is the utility function. Through this formula, we can see that the maximum value of $a_{\text{EI}}$ is the optimum point. On the left side, $u\left( z \right)$ must be reduced as much as possible, and on the right side, the covariance $k\left( {z,z} \right)$ must be increased as much as possible.

To avoid obtaining the local minimum value of the objective function, the positive acquisition functions modify their behavior based on whether they estimate that they are overexploiting an area. Assuming $\sigma F\left( z \right)$ is the standard deviation of the posterior objective function at position $z$, the posterior standard deviation of the additive noise is $\sigma$, and $t_{\sigma }$ is the value of the exploration ratio option, which is a positive number. After each iteration, the positive acquisition functions evaluate whether the next point $z$ satisfies:

11 $$\sigma F\left( z \right) < \sigma t_{\sigma } .$$

If this condition is satisfied, the algorithm will consider point $z$ an overexploited point. Then, the acquisition function modifies its kernel function by multiplying $\theta$ by the number of iterations [33]. Then, a new point is generated according to the new fitted kernel function. Evaluating the new point $z$, if it is again overexploited, $\theta$ is multiplied by an additional factor of 10, and the above steps are repeated. This continues up to five times in an attempt to obtain a point $z$ that is not overexploited as the next point.

### Filter enhancement

Each aneurysm corresponds to a set of optimal filter detection parameters that can be found by Bayesian optimization. When the minimum loss function is found, the corresponding parameters constitute the aneurysm detection parameters, which are substituted into the filter for sphere region enhancement, and the enhanced region is designated the detection target.

### Adaptive thresholding

In this paper, two parameters were used to determine whether the detected target was an aneurysm: one was the maximum value ($V_{\hbox{max} }$) of the filtering response of the detected target, and the other was the mean value ($V_{\text{mean}}$) of the filtering response of the detected target. They were defined as follows:

12 $$V_{\hbox{max} } = { \hbox{max} }\left( {\text{\O }} \right),$$

13 $$V_{\text{mean}} = {\text{mean}}\left( {\text{\O }} \right),$$

where ${\text{\O }}$ denotes the set of filter response values of the detected target. If $V_{\text{mean}}$ or $V_{\hbox{max} }$ was greater than a certain threshold, the target was treated as an aneurysm; otherwise, it was not.

### Aneurysm extraction

The detected target was extracted by region growth. First, the position of the maximum filtering response value in the image was located. We used this position as the seed point for region growth, which began in the 3D image space with 26 neighborhoods, and new points were added to the seed region until the final target area was obtained. Because the detected target incompletely coincided with the aneurysm, we first compared the length, width and height of the target extracted from the growth region and then selected their maximum value. Since some aneurysms were not completely spherical, we found the center of mass of the detected target and used this maximum value as the radius to extract the spherical target; if the target was an aneurysm, it was removed.

When a target was removed, the same steps were used in the remaining image until no further aneurysms were detected. It is important to note that to avoid detecting the same target, the detected region had to be removed after each iteration.

## Data Availability

Not applicable.

## Abbreviations

MRA: Magnetic resonance angiography
SAH: Subarachnoid hemorrhage
CTA: Computed tomography angiography
DSA: Digital subtraction angiography
LSTM: Long short-term memory
LDF: Linear discriminant function
CAD: Computer-aided detection
POI: Points of interest
MIP: Maximum intensity projection
AUC: Area under curve
ROC: Receiver operator characteristic
PR: Precision–recall
